# Prevalence and Factors Associated with Eating Disorders in Military First Line of Defense against COVID-19: A Cross-Sectional Study during the Second Epidemic Wave in Peru

**DOI:** 10.3390/ijerph20042848

**Published:** 2023-02-06

**Authors:** Mario J. Valladares-Garrido, Darwin A. León-Figueroa, Cinthia Karina Picón-Reátegui, Abigaíl García-Vicente, Danai Valladares-Garrido, Virgilio E. Failoc-Rojas, César Johan Pereira-Victorio

**Affiliations:** 1 South American Center for Education and Research in Public Health, Universidad Norbert Wiener, Lima 15046, Peru; 2 Oficina de Epidemiología, Hospital Regional Lambayeque, Chiclayo 14012, Peru; 3 Emerge, Emerging Diseases and Climate Change Research Unit, School of Public Health and Administration, Universidad Peruana Cayetano Heredia, Lima 15013, Peru; 4 School of Medicine, Universidad de San Martín de Porres, Chiclayo 14012, Peru; 5 School of Medicine, Universidad Nacional de Piura, Piura 20002, Peru; 6 Sociedad Científica de Estudiantes de Medicina de la Universidad Nacional de Piura (SOCIEMUNP), Piura 20002, Peru; 7 School of Medicine, Universidad Cesar Vallejo, Piura 20001, Peru; 8 Unidad de Epidemiología y Salud Ambiental, Hospital de Apoyo II Santa Rosa, Piura 20008, Peru; 9 Research Unit for Generation and Synthesis Evidence in Health, Universidad San Ignacio de Loyola, Lima 15024, Peru; 10 School of Medicine, Universidad Continental, Lima 15046, Peru

**Keywords:** eating disorders, COVID-19, eating symptoms, military, Peru

## Abstract

Few studies have evaluated eating disorders in military personnel engaged in defense activities during the COVID-19 pandemic. We aimed to determine the prevalence and factors associated with eating disorders in military personnel from Lambayeque, Peru. A secondary data analysis was performed among 510 military personnel during the second epidemic wave of COVID-19 in Peru. We used the Eating Attitudes Test (EAT-26) to assess eating disorders. We explored associations with insomnia, food insecurity, physical activity, resilience, fear to COVID-19, burnout syndrome, anxiety, depression, post-traumatic stress and selected sociodemographic variables. Eating disorders were experienced by 10.2% of participants. A higher prevalence of eating disorders was associated with having 7 to 12 months (PR: 2.97; 95% CI: 1.24–7.11) and 19 months or more (PR: 2.62; 95% CI: 1.11–6.17) working in the first line of defense against COVID-19, fear of COVID-19 (PR: 2.20; 95% CI: 1.26–3.85), burnout syndrome (PR: 3.73; 95% CI: 1.90–7.33) and post-traumatic stress (PR: 2.97; 95% CI: 1.13–7.83). A low prevalence of eating disorders was found in the military personnel. However, prevention of this problem should be focused on at-risk groups that experience mental health burdens.

## 1. Introduction

Eating disorders (ED) are conditions that alter eating habits [1], damaging physical-mental health and interpersonal relationships [2]. They manifest as anorexia nervosa (AN) and bulimia nervosa (BN), among others [1,2]. There are several conditioning factors for the onset of ED in the military population, such as military-related trauma and exposure [3,4], post-traumatic stress disorder, depression and substance dependence (alcohol and tobacco) [5]. Worldwide, the estimated pre-pandemic prevalence of ED was 0.91%, particularly AN (0.16%), BN (0.63%) and binge eating disorder (BED) (1.93%), relatively lower than previously reported due to modifications in the Diagnostic and Statistical Manual of Mental Disorders (DSM) diagnostic criteria [6]. In the military sector, significant prevalence figures ranging from 5 to 8% in women and 0.1% in men have been reported [7].

Following the impact of COVID-19 in physical and mental health [8,9,10,11], cases of ED increased due to social restrictions, dietary disturbances, sleep problems and difficulty in accessing medical care [12,13,14,15,16,17,18,19,20]. To mitigate the effects of the pandemic, governments joined forces with the armed forces [21], a group highly vulnerable to behavioral problems due to COVID-19 [22]. Even before, binge eating (21.8%) had already been reported as the main ED, along with risk factors for developing ED due to the demanding weight regimen, such mental disorders (insomnia, anxiety, depression) and food insecurity [7]. In Peru, the military personnel supported the government lockdown measures as well as the immunization process [23]. Therefore, they were initially exposed to the mental health burden caused by the pandemic. There is, however, limited evidence on the impact of COVID-19 on their eating behaviors, as only the high prevalence of stressors such as nervousness and sadness, which could lead to mental problems, has been reported [24]. Such a scenario hinders the creation of preventive programs and early detection focused on personnel as part of the first line of defense against COVID-19.

Based on the above, the aim of the study is to explore the prevalence and factors associated with eating disorders in military personnel in the Lambayeque region during the COVID-19 health emergency, 2021.

## 2. Materials and Methods

### 2.1. Study Design, Population, and Sample

A secondary analysis of data generated from an analytic cross-sectional observational study was conducted to assess mental health in front-line military personnel in the face of COVID-19 during the second pandemic wave. The primary research was conducted from 2 to 9 November 2021 in the region of Lambayeque, Peru, a city heavily hit by the COVID-19 pandemic with high seroprevalence [25] and lethality [26].

The population for this investigation included 820 military personnel working in COVID-19 pandemic defense activities in the city of Lambayeque. In the primary study, a sample size of 582 individuals was estimated using an expected prevalence of 12.8% [27], 99% confidence level, precision of 2.5%, and adding 20% of the sample to compensate for possible participant losses and rejections. A higher number of participants (*n* = 710) than required was obtained, representing 86.6% of the study population. The final sample for this analysis included 550 military personnel. Sampling for the primary study was non-probability convenience sampling.

#### 2.1.1. Instruments and Variables

- Eating Disorder symptomatology was the outcome, measured with the Eating Attitudes Test-26 (EAT-26). This test consists of 26 self-report questions assessing general eating behavior and 5 additional questions assessing risk behaviors [28,29,30]. EAT-26 allows the detection of probable eating disorder such as anorexia nervosa, bulimia nervosa and binge eating disorder [31]. Each question has 6 response options with different scoring: 0 points (never, rarely, sometimes); 1 point (often); 2 points (very often); 3 points (always) [28,29]. The total score is the sum of the responses to the 26 items, with question 26 being scored inversely. The higher the score, the higher the risk of anorexia nervosa (AN) or bulimia nervosa (BN) [28,29]. The instrument has 3 subscales: (a) diet, with 13 items on avoidance of fattening foods and concerns about thinness; (b) bulimia and food preoccupation, with 6 items on bulimic behaviors and thoughts about food; and c) oral control, with 7 items on self-control of intake and external pressure to gain weight [28,29]. We used the Spanish version of EAT-26 validated by Gandarillas et al. [32]. The instrument used (EAT-26) has 88.9% sensitivity and 97.7% specificity [33]. The EAT-26 is a useful instrument for assessing risk of eating disorder, but it does not provide a definitive diagnosis [34]. A score of 20 or more obtained from the EAT-26 was considered as positive eating disorder symptomatology, which requires further clinical evaluation by mental health professionals [35]. For this study, the Cronbach’s alpha coefficient was 0.93.
- Insomnia was measured with the Insomnia Severity Index (ISI). It consists of seven self-report items that measure the perceived severity of insomnia through a Likert-type scale from 0 to 4 points and a final score from 0 to 28 points. Higher scores reflect a greater degree of insomnia, with a cut-off point of 8 points [36]. It has been validated in older adults [37] and the general Spanish-speaking population [38]. For this study, the Cronbach’s alpha coefficient was 0.88.
- Food Insecurity was measured with the Household Food Insecurity Assessment Scale (HFIAS). It consists of nine items on a Likert scale of 1 to 3 points (1, seldom; 2, sometimes; 3, frequently). It has excellent psychometric properties in the Latin American population [39]. It has three domains: (1) anxiety and uncertainty about food supply in the household, (2) food quality and insufficient food intake and (3) physical consequences [39]. Mild IA has a score of 2–3 on the first item, 1–3 on the second item or 1 on the third or fourth item [39]. Moderate IA is defined as a score of 2–3 on the third or fourth item, or 1–2 on the fifth or sixth item [39]. Severe IA is defined as a score of 3 on the fifth or sixth item, or 1–3 on factors seven and eight and nine [39]. For this study, the Cronbach’s alpha coefficient was 0.87.
- Physical Activity was measured with the short version of the International Physical Activity Questionnaire (IPAQ-S). This questionnaire includes 9 items and assesses reported physical activity during the last 7 days. It allows obtaining a weighted estimate of total physical activity from the activities reported per week to classify physical activity into: intense, moderate, mild or inactive [40]. It has been validated in Hispanic communities and applied to Latin American populations [41]. For this study, the Cronbach’s alpha coefficient was 0.64.
- Resilience was measured with the Connor-Davidson Resilience Scale (CD-RISC). This questionnaire consists of 10 questions with a Likert scale of 0–4 points (0 “not at all”, 1 “rarely”, 2 “sometimes”, 3 “often”, 4 “almost always”) [42]. It presents adequate internal consistency and validity in multiple occupational groups, health personnel and general adult populations [43,44,45]. It uses a score of less than 30 to define high resilience and less than 30 for low resilience [42,43,44,45]. For this study, the Cronbach’s alpha coefficient was 0.97.
- Fear of COVID-19 was measured with the Fear of COVID-19 Scale. This questionnaire consists of 7 items with a Likert scale of 1–5 points (1 “strongly disagree”, 2 “disagree”, 3 “neither agree nor disagree”, 4 “agree”, 5 “strongly agree”) that evaluate the degree of fear of COVID-19, whereby a higher score indicates a greater fear of COVID-19 [46]. It has excellent psychometric properties and is considered a solid instrument for evaluation in different languages [47]. It has been validated in Latin and Spanish-speaking populations [48,49]. A cut-off point of 16.5 points has been validated to define fear of COVID [50]. For this study, the Cronbach’s alpha coefficient was 0.94.
- Burnout Syndrome was measured with the Maslach Burnout Inventory. It consists of 22 items with a Likert scale of 0–7 points organized in three dimensions that evaluate emotional exhaustion (9 items), depersonalization (5 items) and personal fulfillment (8 items) [51]. It has been validated in the Latin population [52] with adequate validity and reliability properties (Cronbach’s alpha, 0.87; sensitivity, 86.6%; specificity, 89%) [53]. For this study, the Cronbach’s alpha coefficient was 0.91.
- Anxiety was measured with the Generalized Anxiety Disorder-7 Scale (GAD-7). This instrument consists of 7 questions with a Likert scale of 0–3 points (0 “no day”, 1 “several days”, 2 “more than half of the days, 3 “almost every day”) [54]. It assesses anxiety symptoms during the prior 2 weeks, according to DSM-IV criteria [55]. Scores are grouped into no anxiety (0–4 points), mild anxiety (5–9 points), moderate anxiety (10–14 points) and severe anxiety (15–21 points). Its psychometric properties are optimal (Cronbach’s alpha, 0.93; sensitivity, 86.8%; and specificity, 93.4%) [54]. For this study, the Cronbach’s alpha coefficient was 0.93.
- Depression was measured with the Patient Health Questionnaire-9 (PHQ-9): This questionnaire evaluates the presence of depressive symptoms during the prior 2 weeks and is based on DSM-IV criteria [56]. It presents 9 items and uses a Likert scale from 0 to 3 points to evaluate four response options (0 “never”, 1 “several days”, 2 “more than half of the days”, 3 “almost every day”) and has a final score range between 0 to 27 points. It has been validated in the Peruvian population and shows excellent internal consistency (Cronbach’s alpha: 0.87) [57]. For this study, the Cronbach’s alpha coefficient was 0.92.
- Post-traumatic stress disorder was measured with the PTSD Checklist-Civilian Version (PCL-C): This instrument is made up of 17 questions with a Likert scale of 1–5, which measure symptoms of post-traumatic stress disorder, based on the DSM-IV criteria and the rubric of the National Center for PTSD [58,59]. It comprises the domains of trauma re-experiencing (domain B), trauma avoidance and blunting (domain C) and hyperactivity (domain D) [58,59]. It presents a score from 17 to 85 points, with 43 points being the cut-off point to define PTSD [58,59]. It has been validated in Latin populations [60], demonstrating adequate psychometric properties in its internal validity [59]. In the military population, it has been found to have adequate internal consistency and convergent and discriminant validity [61]. For this study, the Cronbach’s alpha coefficient was 0.95.
- General, occupational and psychosocial data: age in years, gender (male, female), single marital status (no, yes), religion (none, Catholic, non-Catholic), children (no, yes), report of frequent alcohol and tobacco consumption (no, yes), report of comorbidities (arterial hypertension, diabetes), body mass index (underweight, normal, overweight, obese), work time (1 to 6 months, 7 to 12 months, 13 to 18 months, 19 months or more), reported personal prior mental health history (no, yes), reported family prior mental health history (no, yes), sought help for mental health problem during COVID pandemic (no, yes), reliance on government to handle COVID (no, yes).

#### 2.1.2. Procedures

A request was sent to the military authority in Lambayeque, Peru, to interview in person the military personnel who were working in the first line of defense against the COVID-19 health emergency. We used a questionnaire designed in REDCap, a data entry system that allowed us to capture data with optimal quality control. After obtaining the authorization from the ethics committee and military authority, the organization to conduct the study began among the research team. The interviews were scheduled in three groups in two shifts (morning and afternoon), under the supervision of the officer in charge and the research team. The soldiers were gathered in ventilated environments, always ensuring the social distance between each one, hand sanitization with alcohol and mandatory use of masks. The supervisor distributed the questionnaire through a link sent to coordination groups of participating soldiers. Prior to accessing the questionnaires, the participants had to voluntarily agree to participate by means of the electronic informed consent form inserted in the first part of the link. Each military member was autonomous to participate in the study and was not obliged by their superior coordinators to answer the questionnaire.

#### 2.1.3. Statistical Analysis

We downloaded the database from the REDCap data entry system and imported it into Stata 17 (StataCorp, College Station, TX, USA).

In the descriptive analysis, we report absolute and relative frequencies of categorical variables. For numerical variables, we reported the best measure of central tendency and dispersion, after evaluating normal distribution.

We performed bivariate analysis between the outcome (eating disorder symptoms) and covariates, using the chi-square test after evaluating the expected frequency assumption.

We estimated crude and adjusted prevalence ratios (PR) and 95% confidence intervals (95%CI) to explore factors associated with eating disorder symptoms. We used generalized linear Poisson family models with log link function to construct simple and multiple regression models. In the multiple regression model, we included variables whose *p*-value was statistically significant (*p* < 0.05) in the simple model. We evaluated collinearity between the covariates of interest.

#### 2.1.4. Ethical Aspects

Authorization was obtained from Universidad San Martin de Porres to conduct the primary research. The database was anonymized, preserving the confidentiality and anonymity of the military participants. Informed consent was requested, and the ethical principles of the Declaration of Helsinki were respected

## 3. Results

### 3.1. General Characteristics

In the primary study, 86.6% of the population was enrolled (*n* = 710), including military personnel actively working in response to COVID-19 and who gave their consent to participate in the study. For this analysis, military personnel who responded to the EAT-26 questionnaire were considered and 160 military personnel who did not respond to the variables of interest were excluded.

Of 550 participants, 95.5% were male and the median age was 22 years. Frequent consumption of alcohol and tobacco was reported by 17.6% and 6.7%, respectively. Arterial hypertension was reported by 9.6%, and 33.6% reported being overweight. Labor time was reported more frequently around 19 months or more (36.9%). Around a third of participants (33.6%) had overweight and 6.8% obesity. Almost half of participants (48.7%) suffered from food insecurity and 11.6% reported low physical activity. Burnout syndrome and post-traumatic stress disorder were experienced by 9.3% and 7.5% of participants, respectively. Regarding mental health outcomes, 19.2% experienced fear of COVID-19, while 14% and 7.5% had suicidal risk and post-traumatic stress disorder, respectively. Additionally, 10.2% of the military presented eating disorder symptoms (Table 1).

**Figure 1:**
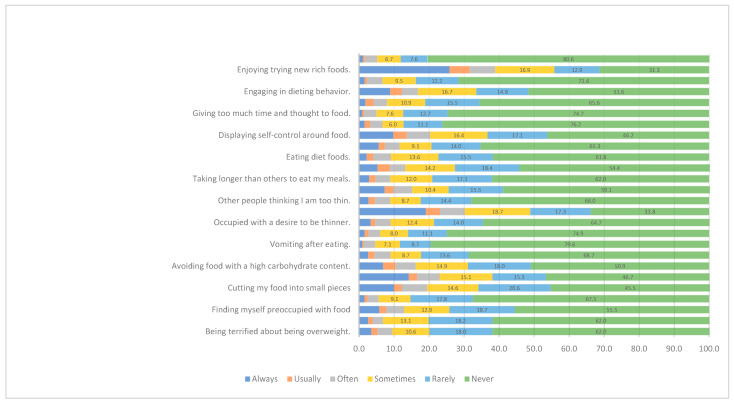
Distribution of responses from the Eating Attitudes Test-26.

In addition, 19.1% of participants reported that they always exercise a lot to burn calories, 14.2% reported that they always consider the calories in the food they eat, and 10% cut their food into small pieces (Figure 1).

### 3.2. Factors Associated with Eating Disorder Symptoms

Labor time (*p* = 0.022), insomnia (*p* < 0.001), fear of COVID-19 (*p* < 0.001) and burnout syndrome (*p* = 0.005) were found to be significantly associated with eating disorder symptoms. Additionally, military members with depressive (*p* = 0.009) and anxious (*p* < 0.001) symptoms had a higher frequency of eating disorder symptoms. Military members with post-traumatic stress disorder had a higher frequency of eating disorder symptoms than those who did not suffer from this mental disorder (*p* < 0.001) (Table 2).

### 3.3. Factors Associated with Eating Disorder Symptoms in Simple and Multiple Regression Analysis

**Figure 2:**
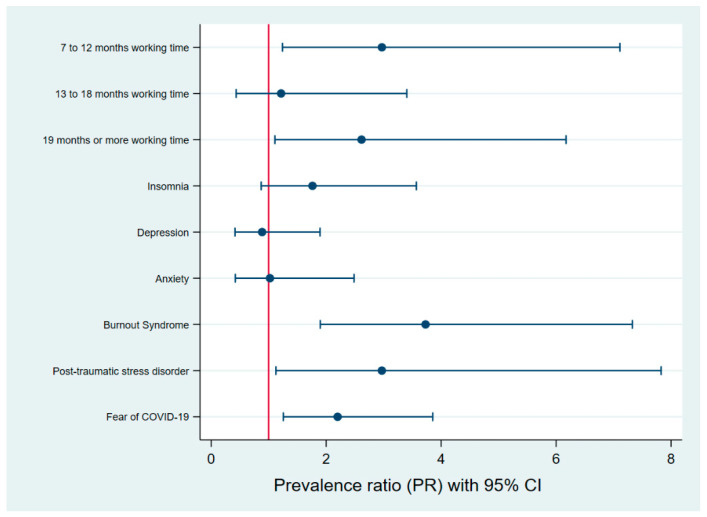
Forest plot of the factors associated with eating behavior in multiple regression analysis.

In the simple model (Table 3), the factors associated with a higher prevalence of eating disorder symptoms were working time, insomnia (PR: 2.71; CI 95%: 1.67–4.42), fear of COVID (PR: 3.36; CI 95%: 2.05–5.49), presenting burnout syndrome (PR: 2.39; CI 95%: 1.32–4.33), anxiety (PR: 2.50; CI 95%: 1.53–4.09), depression (PR: 1.91; PR: 1.17–3.14) and post-traumatic stress (PR: 4.14; CI 95%: 2.47–6.92) (Table 3). In the multiple regression model, we found that military personnel with work time between 7 to 12 months and 19 months or more had 197% (PR: 2.97; CI 95%: 1.24–7.11) and 162% (PR: 2.62; CI 95%: 1.11–6.17) higher prevalence of eating disorder symptoms, respectively. Fear of COVID increased the prevalence of eating disorder symptoms by 120% (PR: 2.20; CI 95%: 1.26–3.85) Additionally, military members with burnout syndrome and post-traumatic stress disorder presented 273% (PR: 3.73; CI 95%: 1.90–7.33) and 197% (PR: 2.97; CI 95%: 1.13–7.83) higher prevalence of eating disorder symptoms, respectively (Figure 2).

## 4. Discussion

### 4.1. Main Findings

Factors associated with a higher prevalence of ED were being at work 19 months longer in the face of the COVID-19 pandemic, fear of COVID-19, having burnout syndrome and having PTSD. Additionally, factors that were not associated with EDs were insomnia, anxiety and depression.

### 4.2. Prevalence of Eating Disorder Symptoms

We found that 1 in 10 military personnel presented eating disorder symptoms (10.2%). This finding is similar to that reported by Masheb et al. in US military veterans in 2021, where 32.8% of women and 18.8% of men evidenced symptoms consistent with a DSM-5 eating disorder diagnosis [62]. In a similar study in a population of military veterans in the United States in 2021, Mitchell et al. found a prevalence of eating disorders ranging from 9.9% to 27.7% [5]. The prevalence found could be explained by the high rates of psychological trauma they face in their military service; strict physical fitness requirements and weight standards; the possibility of being exposed to or witnessing violence; fear of death, dying, and harm to self or others during combat; killing during combat; and changes in eating behavior during service [4,7].

### 4.3. Factors Associated with Eating Disorder Symptoms

Being in service more than 18 months or longer in the face of the COVID-19 pandemic increased the prevalence of eating disorder symptoms. This is similar to that reported by Górska et al. in 2021, where the impact of the COVID-19 pandemic on residents of Europe, Australia, and North and South America increased the prevalence of eating disorders in people who were working [63]. This finding could be explained by the fact that prolonged hours of work on the public road may cause eating disorders because of the military human resource gap in the face of the COVID-19 pandemic [63].

Military personnel with fear of COVID-19 significantly increased the prevalence of conduct disordered eating. Rodgers et al. assert that the COVID-19 pandemic has led to an increase in eating disorders, as fear of COVID-19 transmission has led individuals to experience increased concerns regarding food quality or its ability to be a vehicle for transmission and disruptions in daily routines and activity restrictions have negatively impacted eating and exercise patterns [13]. Fernandez-Aranda et al. reported that confinement due to the COVID-19 pandemic and the uncertainty of becoming infected, has generated in people restrictions in their daily activities, changing their lifestyles and having an impact on the significant increase in eating disorders [64]. This finding could be explained by the fear of becoming infected with SARS-CoV-2 through food, due to the high rate of transmission and mortality resulting from COVID-19 disease evidenced during the second pandemic wave in our country [65,66]. 

We found that military members with burnout syndrome had a higher prevalence of eating disorder symptoms. This is similar to that reported by Özcan et al. in 2021 in Turkey, where having job burnout was associated with a higher frequency of eating disorders, causing higher consumption of fast food, infrequent exercise and higher alcohol consumption [67]. Likewise, Verhavert et al. reported that dietary behavior was positively associated with the risk of burnout syndrome generating in people the consumption of fast food and unhealthy food [68]. This finding could be explained by the fact that military personnel are under constant pressures and desires to optimize performance in their service and pressures to meet certain objectives with traits of compulsiveness and perfectionism, leading to burnout and generating eating disorders [69,70].

Having PTSD increased the prevalence of eating disorders symptoms. In a US study by Vaught et al. in military veterans in 2021, the prevalence of PTSD in patients with eating disorders was found to have a mean of 56.4 [71]. Forman-Hoffman, in 2012, reported in her study a prevalence of PTSD in female veterans with eating disorders of 18.6% [72]. This finding could be explained because PTSD and eating disorders share common symptoms such as emotion dysregulation, impulsivity and alexithymia [73], thus people with PTSD choose a higher intake of unhealthy foods and/or a lower intake of healthy foods generating an increase in eating disorders [74].

In the raw model, it was evidenced that having anxiety and depression is positively associated with eating disorder symptoms. However, this association was not maintained in the final model. This differs from what was reported by Elran-Barak et al. in their study conducted in adults from the Project on Implicit Mental Health (PIMH) website, who found a positive association between eating disorder and mental health (depression and anxiety), and 10.2% and 20.9% presented depressive and anxious symptoms, respectively [75]. This is different from that reported by Sander et al. who found that anxiety and depression were associated with a higher frequency of ED, and 81.1%, 16.6% and 12.5% of people had eating disorders, anxiety and depression, respectively [76]. This finding could be explained by interruptions in access to food and eating routines and movement and exercise restrictions causing a higher level of anxiety and depression in the population [77]. 

### 4.4. Public Health Implications of Findings

The international outbreak of coronavirus disease (COVID-19) has led countries to implement drastic containment measures, suggesting an abrupt confinement of populations causing addiction-related habits, such as caloric/salty food intake [78]. Eating disorders are a public health problem, have high mortality rates and are among the most costly disorders to treat [7], thus bringing serious consequences for the psychological and physical health of the population [71]. However, little is known about the prevalence of such conditions in the military population [7]. In the face of the COVID-19 outbreak, the military were responsible for creating social distance, maintaining quarantine, disinfecting city streets, and establishing field hospitals, forming part of the first line of care against COVID-19 together with health personnel [79].

There are several potential risk factors for the development of ED among military service members, who may engage in disordered eating behaviors, including self-induced vomiting and taking laxatives, diuretics, and diet pills, in response to the strict physical fitness and weight standards imposed by the military [7]. 

Patients with eating disorders constitute a vulnerable population in the context of the COVID-19 pandemic [80], which can be reduced by early and appropriate help-seeking; however, despite the availability of effective treatments, very few people with eating disorders seek treatment [81].

### 4.5. Limitations and Strengths

The study has several limitations. First, the cross-sectional design does not allow causality to be inferred. The second is nonresponse bias, as military personnel could experience very sensitive variation in the degree of motivation in their voluntary participation in the study, either by over- or under-reporting. The third is membership bias among study participants, where subgroups are present due to their hierarchical levels, which makes them share some attribute, which could be positively or negatively related to the outcomes. The fourth is information bias, since the variables were measured by self-report of the participants. In addition, the findings of this research should be interpreted with some caution given that the EAT-26 instrument provides risk assessment of eating disorder and not a definitive diagnosis; therefore it requires an evaluation by a medical professional [35,82]. Fifth, some variables such as race [5], sexual disorder (sexual harassment and assault) [83], addiction to social networks [35], self-esteem [35] and body satisfaction [35] could not be measured and would be associated with eating disorder, and this is because it is a secondary data analysis. The sixth is selection bias, since our population was entirely from the Lambayeque region; considering that there are many military personnel from other regions of Peru, our results could not be extrapolated to other regions due to systematic differences with our population. However, this study provides valuable information on the eating behavior of the military population when exposed to COVID-19; to our knowledge, it is the first evaluation of eating disorders in Peruvian military personnel during the pandemic. Therefore, the results obtained have been carefully inferred and written to draw conclusions that serve as background for future research.

## 5. Conclusions

Almost one out of ten participants experienced eating disorder symptoms. The factors associated with a higher prevalence of eating disorder symptoms were being at work 19 months before the COVID-19 pandemic, fear of getting COVID-19, having burnout syndrome, and having PTSD. Special attention should be given to these factors since the mental health burden of the pandemic may last longer for those vulnerable personnel. This information would be of value for local military institutions and provide feedback for future research in this topic.

## Figures and Tables

**Table 1:** Characteristics of participants (*n* = 550).

Characteristics		*n* (%)
Age (years) *		22 (19–32)
Gender		
	Female	25 (4.6)
	Male	525 (95.5)
Single		
	No	147 (26.7)
	Yes	403 (73.3)
Religion		
	None	82 (14.9)
	Catholic	379 (68.9)
	Non-Catholic	89 (16.2)
Children		150 (27.3)
Alcoholism		97 (17.6)
Smoking		37 (6.7)
Comorbidity		
	Hypertension	53 (9.6)
	Diabetes	10 (1.8)
BMI (categorized) †		
	Underweight/Normal	323 (59.6)
	Overweight	182 (33.6)
	Obesity	37 (6.8)
Personal mental health history		
	No	543 (98.7)
	Yes	7 (1.3)
Family mental health history		
	No	525 (95.5)
	Yes	25 (4.6)
Seeking mental health help		
	No	504 (91.6)
	Yes	46 (8.4)
Trust in government to handle COVID-19		
	Yes	299 (54.4)
	No	251 (45.6)
Labor time†		
	1 to 6 months	138 (25.7)
	7 to 12 months	85 (15.8)
	13 to 18 months	116 (21.6)
	19 months or more	198 (36.9)
Insomnia		
	No	424 (77.1)
	Yes	126 (22.9)
Food insecurity		
	No	282 (51.3)
	Yes	268 (48.7)
Physical activity		
	Low	64 (11.6)
	Moderate	39 (7.1)
	High	447 (81.3)
Resilience		
	Low	311 (56.6)
	High	239 (43.5)
Fear of COVID-19 †		
	No	424 (80.8)
	Yes	101 (19.2)
Burnout Syndrome		
	No	499 (90.7)
	Yes	51 (9.3)
Anxiety		
	No	430 (78.2)
	Yes	120 (21.8)
Depression		
	No	387 (70.4)
	Yes	163 (29.6)
Suicidal risk †		
	No	442 (86.0)
	Yes	72 (14.0)
Post-traumatic stress disorder		
	No	509 (92.6)
	Yes	41 (7.5)
Eating disorder symptoms		
	No	494 (89.8)
	Yes	56 (10.2)

* Median (25th percentile–75th percentile). † Some values may not add up to 550 due to missing data.

**Table 2:** Factors associated with eating disorder symptoms in bivariate analysis.

Variables		*Eating Disorder Symptoms*		p *
		No (*n* = 494)	Yes (*n* = 56)	
		*n* (%)	*n* (%)	
Age (years) ***		22 (19–32)	22 (19–32)	0.626 **
Gender				0.325
	Female	21 (84.0)	4 (16.0)	
	Male	473 (90.1)	52 (9.9)	
Single				0.758
	No	133 (90.5)	14 (9.5)	
	Yes	361 (89.6)	42 (10.4)	
Religion				0.728
	None	73 (89.0)	9 (11.0)	
	Catholic	339 (89.5)	40 (10.6)	
	Non-Catholic	82 (92.1)	7 (7.9)	
Children		135 (90.0)	15 (10.0)	0.931
Alcoholism		86 (88.7)	11 (11.3)	0.678
Smoking		30 (81.1)	7 (18.9)	0.069
Comorbidity				
	Hypertension	48 (90.6)	5 (9.4)	0.850
	Diabetes	8 (80.0)	2 (20.0)	0.300
BMI (categorized)				0.281
	Underweight/Normal	296 (91.6)	27 (8.4)	
	Overweight	159 (87.4)	23 (12.6)	
	Obesity	34 (91.9)	3 (8.1)	
Personal mental health history				0.105
	No	489 (90.1)	54 (9.9)	
	Yes	5 (71.4)	2 (28.6)	
Family mental health history				0.295
	No	470 (89.5)	55 (10.5)	
	Yes	24 (96.0)	1 (4.0)	
Seeking mental health help				0.728
	No	452 (89.7)	52 (10.3)	
	Yes	42 (91.3)	4 (8.7)	
Trust in government to handle COVID-19				0.330
	Yes	272 (91.0)	27 (9.0)	
	No	222 (88.5)	29 (11.6)	
Labor time				**0.022**
	1 to 6 months	132 (95.7)	6 (4.4)	
	7 to 12 months	71 (83.5)	14 (16.5)	
	13 to 18 months	105 (90.5)	11 (9.5)	
	19 months or more	174 (87.9)	24 (12.1)	
Insomnia				**<0.001**
	No	393 (92.7)	31 (7.3)	
	Yes	101 (80.2)	25 (19.8)	
Food insecurity				0.935
	No	253 (89.7)	29 (10.3)	
	Yes	241 (89.9)	27 (10.1)	
Physical activity				0.856
	Low	57 (89.1)	7 (10.9)	
	Moderate	36 (92.3)	3 (7.7)	
	High	401 (89.7)	46 (10.3)	
Resilience				0.448
	Low	282 (90.7)	29 (9.3)	
	High	212 (88.7)	27 (11.3)	
Fear of COVID-19				**<0.001**
	No	394 (92.9)	30 (7.1)	
	Yes	77 (76.2)	24 (23.8)	
Burnout Syndrome				**0.005**
	No	454 (91.0)	45 (9.0)	
	Yes	40 (78.4)	11 (21.6)	
Anxiety				**<0.001**
	No	397 (92.3)	33 (7.7)	
	Yes	97 (80.8)	23 (19.2)	
Depression				**0.009**
	No	356 (92.0)	31 (8.0)	
	Yes	138 (84.7)	25 (15.3)	
Suicidal risk				0.810
	No	397 (89.8)	45 (10.2)	
	Yes	64 (88.9)	8 (11.1)	
Post-traumatic stress disorder				**<0.001**
	No	467 (91.8)	42 (8.3)	
	Yes	27 (65.9)	14 (34.2)	

* *p*-value of categorical variables calculated with the Chi-square test. ** *p*-value of categorical variables-numerical calculated with the U test (Mann–Whitney). *** Median-interquartile range.

**Table 3:** Factors associated with eating disorder symptoms in military first line of defense against COVID-19 in Lambayeque, Peru in simple and multiple regression analysis.

Characteristics		*Eating Disorder Symptoms*					
		Simple Regression			Multiple Regression		
		PR	CI 95%	*p* *	PR	CI 95%	*p* *
Age (years)		1.01	0.98–1.03	0.535			
Gender							
	Female	Ref.					
	Male	0.62	0.24–1.58	0.315			
Single							
	No	Ref.					
	Yes	1.09	0.62–1.94	0.759			
Religion							
	None	Ref.					
	Catholic	0.96	0.49–1.90	0.911			
	Non-Catholic	0.72	0.28–1.84	0.488			
Children		0.98	0.56–1.71	0.931			
Alcoholism		1.14	0.61–2.13	0.677			
Smoking		1.98	0.90–4.37	0.091			
Comorbidity							
	Hypertension	0.92	0.38–2.20	0.851			
	Diabetes	2.00	0.56–7.10	0.283			
BMI (categorized)							
	Underweight/Normal	Ref.					
	Overweight	1.51	0.89–2.56	0.124			
	Obesity	0.97	0.31–3.05	0.958			
Personal mental health history							
	No	Ref.					
	Yes	2.87	0.87–9.53	0.085			
Family mental health history							
	No	Ref.					
	Yes	0.38	0.05–2.65	0.330			
Seeking mental health help							
	No	Ref.					
	Yes	0.84	0.32–2.23	0.730			
Trust in government to handle COVID-19							
	Yes	Ref.					
	No	1.28	0.78–2.10	0.331			
Labor time							
	1 to 6 months	Ref.			Ref.		
	7 to 12 months	3.79	1.51–9.49	**0.004**	2.97	1.24–7.11	**0.015**
	13 to 18 months	2.18	0.83–5.72	0.113	1.22	0.44–3.40	0.708
	19 months or more	2.79	1.17–6.65	**0.012**	2.62	1.11–6.17	**0.028**
Insomnia							
	No	Ref.			Ref.		
	Yes	2.71	1.67–4.42	**<0.001**	1.76	0.87–3.57	0.115
Food insecurity							
	No	Ref.					
	Yes	0.98	0.60–1.61	0.935			
Physical activity							
	Low	Ref.					
	Moderate	0.70	0.19–2.56	0.594			
	High	0.94	0.44–1.99	0.874			
Resilience							
	Low	Ref.					
	High	1.21	0.74–1.99	0.449			
Fear of COVID-19							
	No	Ref.			Ref.		
	Yes	3.36	2.05–5.49	**<0.001**	2.20	1.26–3.85	**0.006**
Burnout Syndrome							
	No	Ref.			Ref.		
	Yes	2.39	1.32–4.33	**0.004**	3.73	1.90–7.33	**<0.001**
Anxiety							
	No	Ref.			Ref.		
	Yes	2.50	1.53–4.09	**<0.001**	1.02	0.42–2.49	0.960
Depression							
	No	Ref.			Ref.		
	Yes	1.91	1.17–3.14	**0.010**	0.89	0.42–1.89	0.756
Suicidal risk							
	No	Ref.					
	Yes	1.09	0.54–2.22	0.809			
Post-traumatic stress disorder							
	No	Ref.			Ref.		
	Yes	4.14	2.47–6.92	**<0.001**	2.97	1.13–7.83	**0.028**

* *p*-values obtained with generalized linear models, Poisson family, log link function, robust variance.

## Data Availability

The dataset generated and analyzed during the current study is not publicly available because the ethics committee has not provided permission/authorization to publicly share the data, but the data are available from the corresponding author on reasonable request.
